# Severe Facet Joint Arthrosis Caused C7/T1 Myelopathy: A Case Report

**DOI:** 10.1155/2009/481459

**Published:** 2009-06-16

**Authors:** Toshimi Aizawa, Hiroshi Ozawa, Takeshi Hoshikawa, Takashi Kusakabe, Eiji Itoi

**Affiliations:** Department of Orthopaedic Surgery, Tohoku University School of Medicine, Sendai 980-8574, Japan

## Abstract

Cervical myelopathy is caused by degenerative processes of the spine including intervertebral disc herniation and posterior spur usually developing at C3/4 to C5/6. C7/T1 single level myelopathy is very rare because of the anatomical characteristics. Facet joint arthrosis can be a cause of cervical myelopathy but only a few cases have been reported. The authors report an extremely rare case of C7/T1 myelopathy caused by facet joint arthrosis. A 58-year-old male presented with hand and gait clumsiness. The radiological examinations revealed severe C7/T1 facet joint arthrosis with bony spur extending into the spinal canal, which compressed the spinal cord laterally. The T1 spinous process indicated nonunion of a “clay-shoveler's” fracture, which suggested that his cervico-thoracic spine had been frequently moved, and thus severe arthrosis had occurred in the facet joints. A right hemilaminectomy of C7 and C7/T1 facetectomy with single level spinal fusion led to complete neurological improvement.

## 1. Introduction

Cervical myelopathy results from compression of the spinal cord and can cause motor and sensory dysfunction of the upper and lower extremities as well as bladder dysfunction [1]. In 1988, the Department of Orthopaedic Surgery, Tohoku University School of Medicine, and its affiliated hospitals started a registration system of all spine surgeries in Miyagi prefecture and its surrounding area, in the northeastern Japan [1–6]. More than 38 000 patients were registered in the past 20 years, and about 20% of the patients were operated because of cervical myelopahty caused by various degenerative processes of the spine [6]. The pathogenesis of these myelopathy patients was basically divided into 7 categories: developmental stenosis, dynamic stenosis, disc herniation, segmental ossification of the posterior longitudinal ligament (OPLL), continuous OPLL, posterior spur rising from the vertebral bodies and uncinate processes, and calcification of the ligamentum flavum (CLF) [1]. In addition, there were rare patients with minor pathogeneses such as anterolisthesis and degenerative scoliosis [7, 8]. 

 The most common symptomatic disc level of cervical myelopathy caused by degenerative processes of the spine is C5/6 and C4/5, followed by C3/4 [1, 9]. Occasionally, we can see patients whose spinal cord at C7/T1 is compressed by OPLL spread over from cervical and thoracic spines. However, single level C7/T1 myelopathy is rare. Only a few reports have described this myelopathy as caused by the ossification of the ligamentum flavum (OLF) and degenerative anterolisthesis [2, 10–12]. 

 Facet joint arthrosis or arthrotic hypertrophy of the facet joints can also be a cause of cervical myelopathy [13]; however, it is rare. Epstein et al. [14] reported 5 cases at C3/4 and C4/5, and Benitah et al. [15] also reported one case at C1/2. To our knowledge, there have been no reports of myelopathy led by facet joint arthrosis in the cervico-thoracic spine. Here, we report the first case of this myelopathy at C7/T1, which was successfully treated by surgical decompression. 

 The patient was informed that his data would be submitted for publication and gave consent.

## 2. Case Report

A 58-year-old male carpenter first noticed pain in the ulnar side of both arms in October, 2006. He had no history of trauma in the cervical and upper thoracic regions. Since February 2007, he felt clumsiness in both hands, and his gait was disturbed so that came to our clinic in May 30. 

### 2.1. Neurological Examination

His gait was slightly spastic, and he had pollakiuria. Neurological examination revealed muscle weakness grade G in the bilateral wrist flexors, finger extensors, and iliopsoas muscles and grade F in the bilateral finger abductors. The triceps tendon reflex was decreased while the knee and ankle jerks were accelerated bilaterally. Sensory disturbance was detected on the ulnar side of both arms, on the C8 dermatome, and on and below the L1 dermatomes. We diagnosed cervical myelopathy at the C6/7 spinal level or perhaps at the C7/T1 level although the latter would be very rare. The Japanese Orthopaedic Association (JOA) score for cervical myelopathy was 8 of 17 [16].

### 2.2. Radiological Findings

**Figure 1:**
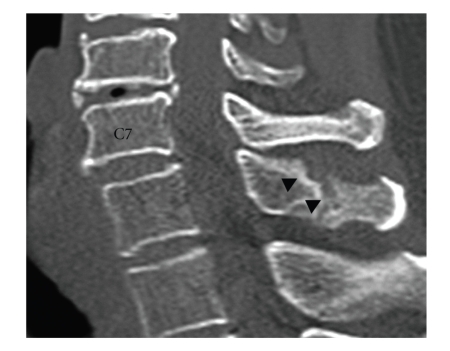
A sagittal reconstructed computed tomogram (CT). Nonunion of the T1 spinous process fracture (arrowheads) and slight anterolisthesis of C7/T1 are detected.

**Figure 2:**
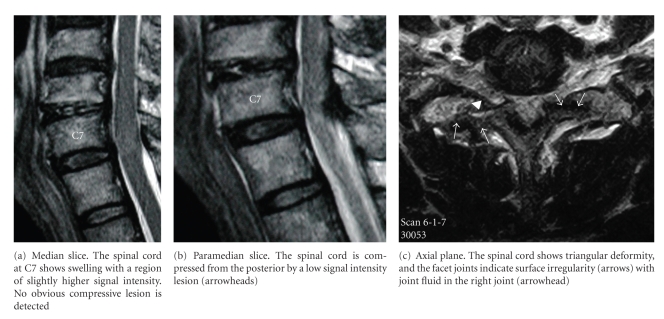
MRI on T2-weighted image.

**Figure 3:**
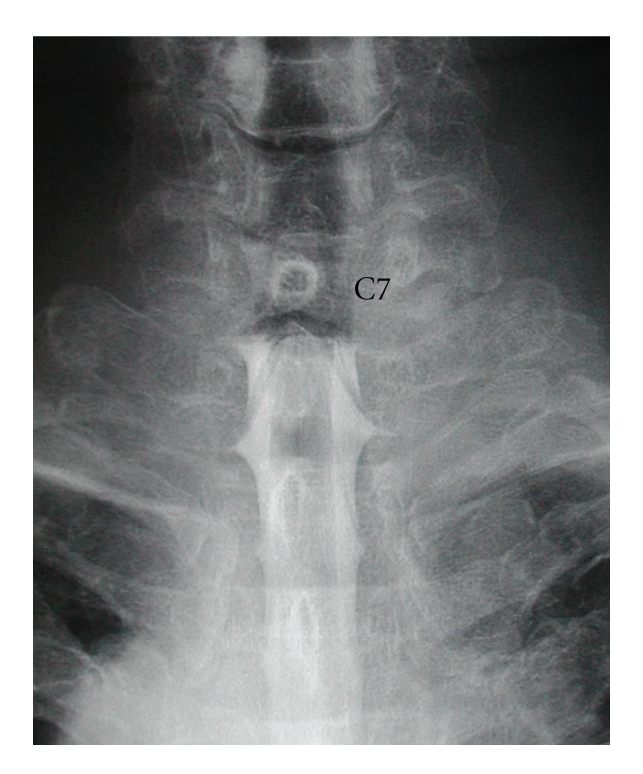
Postero-anterior view of myelogram. The contrast medium stops completely at C7/T1 disc level.

**Figure 4:**
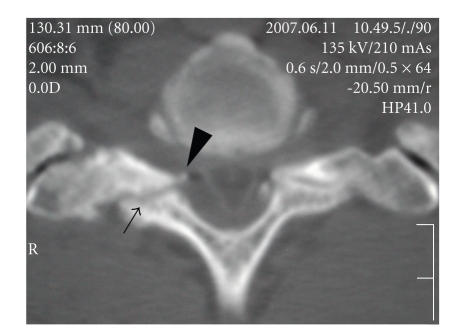
Computed tomographic myelography at C7/T1 level. The spinal cord is compressed from both sides and shows atrophy. The right C7/T1 facet joint indicates severe arthrosis (arrow) with bony spur extending into the spinal canal (arrowhead).

Plain lateral radiographs showed no spinal canal stenosis in the cervical spine. A sagittal reconstructed computed tomogram (CT) indicated nonunion of the T1 spinous process fracture and slight anterolisthesis of C7 (Figure 1). In the sagittal planes of magnetic resonance imaging (MRI), the spinal cord at C7/T1 showed swelling with a slightly higher signal intensity region on the T2-weighted images in the median slice, although no compressive factors were detected. On the other hand, the cord at this level was compressed from posterior in the paramedian slices. The axial planes of MRI demonstrated triangular deformity of the spinal cord and surface irregularity of the facet joints (Figure 2). A postero-anterior myelogram showed complete block of the contrast medium at C7/T1 (Figure 3). Computed tomographic myelography clearly indicated a spinal cord deformity in an antero-posterior direction and severe arthrosis with bony spur formation from the bilateral C7/T1 facet joints growing into the spinal canal, which was right side dominant (Figure 4). The facet joints in the other spinal levels did not show as severe arthrotic changes as seen in C7/T1.

### 2.3. Operation

From the neurological and radiological findings, we diagnosed that the patient had a cervical myelopathy at C7/T1, which was mainly caused by facet joint arthrosis. On August 7, his spinal cord was decompressed through a hemilaminectomy of C7 and C7/T1 facetectomy in the right side. Then, interlaminar wiring described by Rogers W.A. and left facet joint fusion with iliac bone graft were added for the spinal stability of C7/T1 [17].

### 2.4. Pathological Findings

The histological examination revealed that the resected area consisted of cartilage, bone, and synovium. No inflammation was detected. These findings were consistent with the interpretation that the specimen involved an arthropathized facet joint.

### 2.5. Postoperative Course

**Figure 5:**
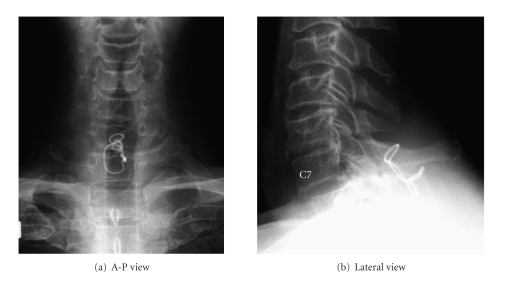
Radiographs one year postoperatively.

Four months after surgery, the patient showed excellent neurological improvement with no motor, sensory, and bladder dysfunctions. His JOA score improved to 17 of 17 and was maintained at 1-year follow-up. Facet fusion was completed 1 year postoperatively, and no instability was detected in C7/T1, as confirmed by dynamic flexion-extension radiographs (Figure 5).

## 3. Discussion

C7/T1 has several anatomical features that differ from other levels of the cervical spine. Except for C1/2, C7/T1 shows the smallest range of motion in all directions: flexion/extension is 9°, one side bending 4°, and rotation 2°, which leads to minor spondylotic changes in the intervertebral disc and facet joints at this spinal level [18]. C7/T1 has a low frequency of the posterolisthesis, posterior spur formation, and intervertebral disc herniation. Unlike the intervertebral spaces in the other cervical levels, C7/T1 lacks Luschka joints. Thus, the C7/T1 intervertebral disc is more likely to herniate laterally, which might cause radiculopathy rather than myelopathy [19, 20]. In addition, the spinal cord has smaller area below the intumenscentia cervicalis at C4/5 to C5/6 [21]. There have been no reports of the percentage of the C7/T1 myelopathy cases amongst all cervical myelopathy, but it would be expected to be very rare.

As mentioned above, there is small range of motion at C7/T1 and thus, minor spondylotic changes. In the present case, however, the C7/T1 had severe arthrosis of the facet joints and slight anterolisthesis. Additionally, the spinous process of T1 showed signs of a fracture nonuninon. A fracture in the spinous process of the cervicothoracic spine is known as a “clay-shoveler's” fracture [22]. This fracture occurs by the actions of the trapezius, the rhomboideus major and minor, and serratus posterior superior muscles [22]. The patient was a carpenter, and those muscles would have been frequently used in his daily works. The cervicothoracic spine, from which these muscles originated, was also used over and over again everyday, which might have caused the severe spondylotic change in the C7/T1 facet joints.

In cervical myelopathy, most of the compressive spinal lesions locate in the anterior and/or posterior of the spinal canal: the intervertebral disc herniation, OPLL and posterior bony spur of the vertebral bodies in the anterior, and CLF in the posterior. Canal size is also very important and is represented by an antero-posterior diameter including posterolisthesis of the vertebral body [1, 23]. Therefore, the sagittal planes of MRI usually demonstrate the spinal cord compression from anterior and/or posterior. However, in the present case, the spinal cord was compressed only laterally. The MRI of the sagittal plane was very characteristic. The spinal cord swelling was detected at C7/T1, and no evidence of compression of the spinal cord was depicted in the median slice, which might lead to a misdiagnosis of having an intramedullary tumor. Careful investigation of the paramedian slices of the sagittal plane and axial planes was necessary to make a correct diagnosis that the spinal cord was compressed laterally. CT clearly indicated that the compression was caused by facet joint arthrosis with bony spurs growing transversely into the spinal canal.

Several surgical options were considered: laminectomy and facetectomy on one side or on both sides, with or without spinal fusion. Since the main compression was due to facet joint arthrosis, at least half of the facet joint should be resected based on the CT findings. However, facetectomy is the most critical factor in determining the risk of the postoprtative kyphosis [24]. To prevent this possible future deformity, spinal fusion should be added. If we decompressed by laminectomy and facetectomy on both sides, we should add more than two levels of spinal fusion using instrumentation. A less invasive surgery would be better for the patient. Thus, spinal cord decompression was performed only through the right side, which had more severe arthrosis and larger bony spur. Only one level of spinal fusion was carried out using interlaminar wiring and bone graft into the remained facet joint. This surgery resulted in an excellent neurological improvement and also in complete bony fusion at C7/T1 spinal level.
